# Examination of Medical College Admission Test Scores and US Medical Licensing Examination Step 1 and Step 2 Clinical Knowledge Scores Among Students With Disabilities

**DOI:** 10.1001/jamanetworkopen.2021.10914

**Published:** 2021-05-21

**Authors:** Joel Purkiss, Melissa Plegue, Christina J. Grabowski, Michael H. Kim, Sharad Jain, Mark C. Henderson, Lisa M. Meeks

**Affiliations:** 1 Division of Evaluation, Assessment, and Education Research–School of Medicine, Department of Education, Innovation, and Technology, Baylor College of Medicine, Houston, Texas; 2 Department of Family Medicine, The University of Michigan Medical School, Ann Arbor; 3 Department of Medical Education, University of Alabama at Birmingham School of Medicine; 4 Department of Medicine, University of Minnesota Medical School, Minneapolis; 5 Office of Medical Education, Department of Internal Medicine, School of Medicine, University of California, Davis, Sacramento; 6 Office of Admissions and Outreach, School of Medicine, University of California, Davis, Sacramento; 7 Center for a Diverse Healthcare Workforce, School of Medicine, University of California, Davis, Sacramento

## Abstract

This cohort study examines the association between Medical College Admission Test (MCAT) scores, disability status and category, and performance on US Medical Licensing Examination (USMLE) Step 1 and Step 2 CK scores.

## Introduction

An increasing number of medical students are disclosing disabilities,^1^ yet little is known about the association between Medical College Admission Test (MCAT) and US Medical Licensing Examination (USMLE) performance in this population. Previous studies were focused on a single site^3^ and were performed prior to changes in disability law and increases in disability disclosure.^2,3^ Historical literature suggests that students with disabilities (SWD) have lower USMLE pass rates^2^ and lower Step 1 and Step 2 Clinical Knowledge (CK) scores.^3^ Despite recent attention to differential MCAT performance among students with lower socioeconomic status backgrounds and those identifying as races/ethnicities underrepresented in medicine,^4^ disability has been conspicuously absent from these discussions. If the MCAT is to remain a useful tool for assessing the likelihood of success in medical school, data on performance outcomes in diverse cohorts of students are needed.^5^ We examined the association of MCAT scores, disability status and category, and performance on Step 1 and Step 2 CK scores in a multisite, multiyear cohort of SWD who matriculated following amendments to the Americans with Disabilities Act.^6^

## Methods

We conducted a retrospective cohort study of 163 graduating SWD from 11 US medical schools in 2018 and 2019 matched with 2 nondisabled control (NDC) participants by self-reported gender at application and MCAT score, yielding a final sample of 488 students. The University of Michigan Medical School institutional review board approved this study. The requirement for informed consent was waived because data were deidentified. The study followed the American Association for Public Opinion Research (AAPOR) reporting guideline. SWD were dichotomized into 2 groups following previous literature.^3^ The cognitive group included students with psychological, learning, and attention deficit disorders; the noncognitive group included all others. Primary outcome measures included scores on USMLE Step 1 and Step 2 CK. To assess the association between MCAT and USMLE scores, we ran linear mixed models using MCAT, disability group, and the interaction between MCAT and disability group as covariates. Random effects for school and matched pairs were included to account for clustering. Secondary analyses separated SWD into those with cognitive vs noncognitive disabilities and compared each with NDC group participants using a 3-group approach. The NDC group was the reference group for all comparisons. Model assumptions included normality of error terms and random effects; both were assessed and determined to be reasonably met. A significance level of *P* < .05 was used in determining significant associations, and all tests were 2-sided. Statistical analyses were conducted in Stata IC version 15.1 (StataCorp).

## Results

The sample consisted of 488 participants, with 284 (58.2%) female participants and a mean (SD) MCAT score of 31.6 (3.5). Among 163 SWD, 111 (68.1%) reported cognitive disabilities, 47 (28.8%) reported noncognitive disabilities, and disabilities for 5 (3.1%) were unknown. Our models showed that MCAT scores were positively associated with USMLE Step 1 and Step 2 CK scores for all students (Table). After adjusting for MCAT score, SWD status was associated with lower mean USMLE scores (B = −11.2; 95% CI, −14.0 to −8.4). Neither model had a significant disability by MCAT interaction, suggesting that changes in MCAT scores were associated with similar changes in USMLE scores regardless of disability status (Figure). Students with noncognitive disabilities had significantly lower Step 1 scores than students in the NDC group (B = −5.8; 95% CI, −10.6 to −1.1) but did not differ significantly from students in the NDC group on Step 2 CK scores (B = −3.4; 95% CI, −7.7 to 0.9). Students with cognitive disabilities had significantly lower mean scores than both students in the NDC group and those with noncognitive disabilities on both Step 1 (cognitive disability vs NDC: B = −13.3; 95% CI, −16.6 to −10.0; cognitive vs noncognitive disability: B = −7.3; 95% CI, −12.7 to −1.9) and Step 2 CK (cognitive disability vs NDC: B = −10.7; 95% CI, −13.7 to −7.7; cognitive vs noncognitive disability: B = −7.3; 95% CI, −12.2 to −2.4).

**Table.** Association of MCAT Score With US Medical Licensing Examination Step 1 and Step 2 CK Scores for Medical Students With and Without Disabilities

Covariate	Model 1a, Step 1 score^a^		Model 2a, Step 2 CK Score^a^	
	B (95% CI)	*P* value	B (95% CI)	*P* value
MCAT Score	2.27 (1.81 to 2.74)	<.001	1.54 (1.15 to 1.93)	<.001
Group				
Nondisabled control group	0 [Reference]	NA	0 [Reference]	NA
Students with disabilities	−11.20 (−14.04 to −8.36)	<.001	−8.47 (−11.11 to −5.83)	<.001
	**Model 1b: Step 1 score**^**b**^		**Model 2b: Step 2 CK score**^**b**^	
MCAT Score^c^	2.20 (1.72 to 2.68)	<.001	1.46 (1.06 to 1.86)	<.001
Group				
Nondisabled control group	0 [Reference]	NA	0 [Reference]	NA
Students with cognitive disabilities^d^	−13.28 (−16.55 to −10.01)	<.001	−10.71 (−13.74 to −7.68)	<.001
Students with noncognitive disabilities^e^	−5.84 (−10.58 to −1.11)	.02	−3.41 (−7.74 to .93)	.12

Abbreviations: CK, Clinical Knowledge; MCAT, Medical College Admission Test; NA, not applicable.

^a^ Models 1a and 2a include group as control group vs students with disability group.

^b^ Models 1b and 2b include group as a 3-level factor, ie, control group vs cognitive primary disability group vs noncognitive primary disability group.

^c^ Scores were from the previous version of the MCAT exam.

^d^ Cognitive disabilities included psychological, learning, and attention deficit disorders.

^e^ Noncognitive disabilities included mobility/physical disabilities, chronic health conditions, deaf and hard of hearing, and low vision.

**Figure.**
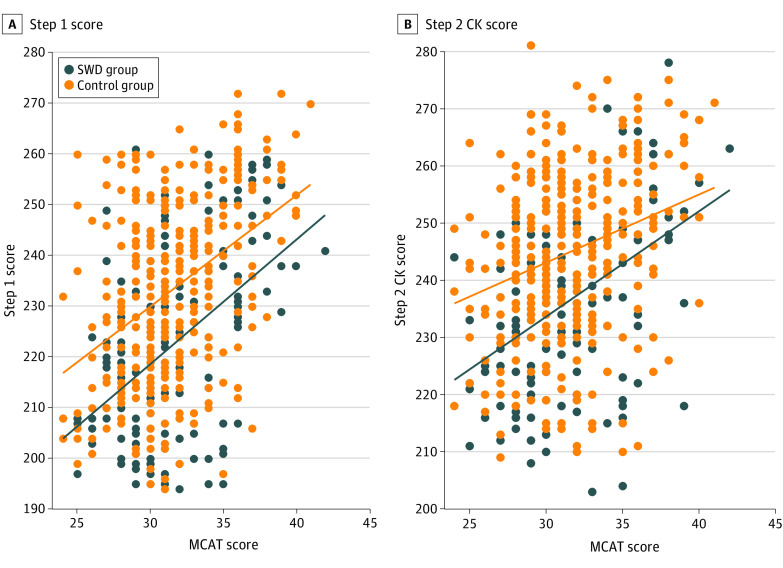
Medical College Admission Test (MCAT) Scores by US Medical Licensing Examination Step 1 and Step 2 Clinical Knowledge (CK) Scores, for Students With and Without Disabilities Lines indicate unadjusted regression fit lines between MCAT and US Medical Licensing Examination scores for students with disabilities (SWD) and the control group.

## Discussion

SWD are a growing and important medical school population. Our findings confirm previous studies^2^ showing that MCAT is strongly associated with USMLE examination scores for both SWD and students without disabilities. This study has several limitations. Dichotomized categorical groups may not fully represent the association of disability with performance. Furthermore, no data on MCAT testing accommodations were available. As the number of SWD grows, understanding the association between MCAT and USMLE scores may better inform admissions committees with concerns regarding the academic performance of applicants with disabilities.
